# The Medical Correspondent

**Published:** 1920-02-28

**Authors:** 


### THE MEDICAL CORRESPONDENT.

A very real and, to those who take genuine and wholehearted interest in matters concerning the public welfare, very urgent question is raised by a recent paragraph in the *Medical Press*, in which the writer, with considerable moderation, both criticises and comments upon the recent activities of medical writers in the lay press. So easily is any paragraph ascribed to "our medical correspondent," and, presumably, so great the degree of trustworthiness thereby apparently ascribed to the attractive dissertations which follow, that we feel there is indeed need, in some quarters at least, for the use of very much greater care in allowing many of these reports to be spread broadcast among the public. As our contemporary points out, since the end of the war, medical features in the daily papers have gained a far more established position, and to judge by appearances the medical contributors are allowed to write as they please, provided they keep to their subject. In exactly what manner and to what degree the public accepts and derives benefit from these mixed explanations and exhortations it is not our object here to discuss. As professional critics we should say that the great majority of the genuine medical articles published are both valuable and well considered. Indeed it is possible to cite more instances than one of famous daily papers which are to be heartily congratulated upon the promptness and wisdom with which they have recently brought the latest medical matters to the fore.

Among the good points, too, there is the modern tendency for local health authorities to make greatly increased use of their local newspapers to bring health propaganda to their charges' notice, causing the publication of many simple straightforward health articles, which undoubtedly meet a far more sympathetic reception when delivered in this form. Here again, to find fault is quite unjustifiable, even if scientific facts are here and there somewhat sketchily treated. Also we even hesitate to criticise the undoubted tendency, obvious to a medical reader at least, for more than a few contributors to assume something of the oracle's pose. But we must remember that the readers of many of these articles, the masses to whom we all wish that advice and enlightment may be given, cannot yet be expected to think medically. There is an American saying, that if you cannot make a man think as you do, then make him do as you think, and in effect that is what the first stages of public medical education must amount to. The layman will take personal advice from a doctor, but inevitably he is not so ready to accept it from the pages of a lay paper, and we think that, what may be termed the personal tone in many of these medical articles, is not utterly to be condemned. It has, where not overdone, many points in its favour. In any case, apparently the habit is most prevalent in those papers which circulate mainly among the poorer classes, the leading dailies being practically exempt. True, that to the professional critic the picture of these apparent medical oracles is amusing, but the good they do may well outweigh the harm. The public has still a lot to learn of the elements of modern hygiene.

On the other hand, we heartily agree with the writer of the note in question when he suggests that "the conductors of many papers would be wise if they submitted in all cases for editorial supervision to the medical man on their staff, the wonderful reports of operations and other medical details often submitted by foreign correspondents, and thus prevent the publication of matter which from the medical aspect is not unfrequently nonsense and misleading." Were this censoring carried out, it would, we fear, deprive more than one of our own contributors of their frequent opportunities to pour forth deserved criticism on these so-called medical "advances," but the public is easily influenced; the present health campaign is no small matter, and the vastest undertakings may be ruined by the smallest initial cause.

